# Event Coverage Detection and Event Source Determination in Underwater Wireless Sensor Networks

**DOI:** 10.3390/s151229875

**Published:** 2015-12-15

**Authors:** Zhangbing Zhou, Riliang Xing, Yucong Duan, Yueqin Zhu, Jianming Xiang

**Affiliations:** 1 School of Computer & Communication Engineering, University of Science & Technology Beijing, Beijing 100083, China; zhangbing.zhou@gmail.com; 2 School of Information Engineering, China University of Geosciences (Beijing), Beijing 100083, China; riliangxing@126.com (R.X.); xiangjianmingzerg@gmail.com (J.X.); 3 Computer Science Department, TELECOM SudParis, Evry 91 011, France; 4 College of Information Science and Technology, Hainan University, Haikou 570228, China; 5 Development Research Center of China Geological Survey, and Key Laboratory of Geological Information Technology, Ministry of Land and Resources, Beijing 100037, China; yueqinzhu@163.com

**Keywords:** event coverage detection, event sources determination, routing tree, weighted graph, underwater wireless sensor networks

## Abstract

With the advent of the Internet of Underwater Things, smart things are deployed in the ocean space and establish underwater wireless sensor networks for the monitoring of vast and dynamic underwater environments. When events are found to have possibly occurred, accurate event coverage should be detected, and potential event sources should be determined for the enactment of prompt and proper responses. To address this challenge, a technique that detects event coverage and determines event sources is developed in this article. Specifically, the occurrence of possible events corresponds to a set of neighboring sensor nodes whose sensory data may deviate from a normal sensing range in a collective fashion. An appropriate sensor node is selected as the relay node for gathering and routing sensory data to sink node(s). When sensory data are collected at sink node(s), the event coverage is detected and represented as a weighted graph, where the vertices in this graph correspond to sensor nodes and the weight specified upon the edges reflects the extent of sensory data deviating from a normal sensing range. Event sources are determined, which correspond to the barycenters in this graph. The results of the experiments show that our technique is more energy efficient, especially when the network topology is relatively steady.

## 1. Introduction

The Earth’s surface is mostly covered by oceans, which impact our life extensively [1]. Although knowledge about the oceans is of core importance to our life, only around 1% of the whole ocean has been explored, due to various factors, including vast volume, high pressure and the harshness of underwater environments [2,3]. The importance and hardness of underwater exploration is evident through the recent search-and-rescue effort for the Malaysia flight MH370 in the Pacific Ocean [4]. In this context, exploring the vast ocean volume has been a critical and urgent task for the last few decades. With the advance of communication and sensing technologies, sensor nodes can be deployed in the underwater environment, and underwater wireless sensor networks (WSNs) have recently attracted significant attention and been considered as a promising alternative to exploring underwater environments [5]. Specifically, smart things, also called underwater sensor nodes, sense and record current (maybe historical, as well) information about underwater environments. These underwater smart things interconnect with each other and establish a network for gathering and routing sensory data to the sink node(s), which is (are) typically sonobuoys deployed on the ocean surface [6]. World-wide underwater WSNs establish the Internet of Underwater Things (IoUT) [7], which aims to collaboratively explore the vast ocean volume. IoUT supports wide-spread applications in scientific, industrial, military and other domains. Sensor nodes in the underwater environment are typically powered by batteries, which are hard, if not impossible, to replace or recharge nowadays [5]. Ambient energy harvesting and batteries with super-capacitors are promising to be adopted in the future [8]. In this setting, energy efficiency is a key factor to be considered, when mechanisms are to be proposed for underwater environment monitoring and event coverage detection [9]. Unlike terrestrial WSNs [10], where sensor nodes can be localized through global positioning systems or other localization techniques [11], sensor nodes underwater are localized through limited communication with anchor nodes or reference nodes, leveraging specific localization technologies [12,13]. Sensory data are gathered and routed to sink node(s) in a pro- or re-active fashion [14,15,16].

Underwater WSNs are mostly responsible for monitoring the oceanic environment and detecting whether events have occurred or not [17]. Generally, an event can be anything that reflects an improper situation according to collective sensory data with respect to certain criteria. An example is presented in [8] about marine shellfish monitoring, where the attributes, like pH value, biotoxin value, water temperature, salinity, *etc*., are monitored. When an event like pollution is detected, sensory data for multiple kinds of attributes should be routed to sink node(s) immediately for quick event coverage detection and proper response enactment. It is worth mentioning that no event may occur in underwater WSNs for most time durations. This means that sensor nodes may not need to report their sensory data to sink node(s), since sensory data may be within the range of healthy. On the other hand, when an improper situation, which may indicate the occurrence of an event, is detected, sensory data of relevant sensor nodes should be gathered and routed to sink node(s) immediately for the identification of the location, coverage and sources for this event. Therefore, energy-efficient techniques for supporting sensory data routing, event location and coverage detection are fundamental in underwater WSNs.

Event coverage detection and location determination have been explored relatively extensively for two- and three-dimensional terrestrial WSNs [18,19,20]. Generally, sensor nodes are either static or moving along pre-determined itineraries and can be localized through global positioning systems or other localization techniques [11]. Inner and outer boundaries are identified for localizing event locations and geographic ranges. It is worth mentioning that sensor nodes are typically deployed very sparsely in underwater WSNs, due to the fact that deploying an underwater WSN is very costly nowadays, and the network topology may change (dramatically) due to the movement of sensor nodes caused by the water dynamics. We argue that these techniques in terrestrial WSNs may hardly be used directly in the context of underwater counterparts. Note that there are some techniques that study event detection and localization in underwater WSNs [17,21]. For instance, virtual sensors are adopted to represent an aggregation point for multiple physical sensors [17], since physical sensors may drift along the water dynamics and, thus, may hardly be localized. Accurate event location is achieved with a relatively low sensing overhead. A monitoring course-based event localization technique is proposed in [21], where monitoring courses can facilitate the event location determination and identify possible network issues before forwarding data packets to sink node(s). Generally, these techniques are promising in determining event boundaries in underwater environments. It is worth noting that the sensory data of sensor nodes, which are internal (or external) to event regions, may not vary dramatically during certain time durations, when the environment to be monitored is relatively steady. Besides, certain applications may tolerate the bias of sensory data to be used and to be sensed in real time to a certain extent. A sample application is marine shellfish monitoring, where the pH value, the biotoxin value, *etc*., may not change to a large extent, when their values have already indicating the occurrence of pollution. In addition, when the value of attributes like pH (or biotoxin) is within a certain range, the pollution is assumed as having the same grade of severity. This means that sensory data gathered at previous time slots may be reused, rather than fetched from the network in real time, for supporting certain applications in the forthcoming time slots, when the value changes slightly. When an event is detected to have occurred, potential event sources should be identified, which should be considered as the key points for the enactment of a prompt and proper response mechanism. Consequently, an energy-efficient mechanism for the detection of event coverage, while considering the reuse of sensory data and accurately determining potential event sources, is a pressing research challenge.

Leveraging our sub-region query processing mechanism developed in our previous work [22] and traditional underwater localization techniques [23], we propose in this article a strategy that aims to detect the event coverage. Our major contributions include the following two aspects: (i) our technique can determine the event coverage in an energy-efficient fashion; and (ii) it can identify potential event sources, which are of importance for prompt and proper response enactment. Without loss of generality, the geographical space of an underwater WSN is defined as a three-dimensional rectangular region. The procedure for this technique includes the following steps:

- Our sub-region query processing mechanism developed in [22] has been improved, where a set of neighboring sensor nodes, whose sensory data deviate from a normal sensing range in a collective fashion, are identified. These sensory data are routed to sink node(s) through our routing tree [22] in an energy-efficient fashion.
  Note that an event is different from an error, which typically corresponds to an outlier in the network [19,24]. Sensory data of errors are excluded from being routed to sink node(s).
- Based on the sensory data of sensor nodes in possible event regions, the coverage of events is detected, which is represented as a network (or a weighted graph) of sensor nodes. Potential event sources are determined through an algorithm that identifies barycenters in a weighted graph [25]. Generally, an event source can be identified as a barycenter in the graph of sensor nodes, whose sensory data deviate the most in value with respect to a normal sensing range.
- Extensive simulations have been conducted to evaluate the effectiveness and efficiency of our event coverage detection and event source determination mechanisms. The results show that our technique is more energy efficient, especially when the network topology is relatively steady.

The rest of this paper is organized as follows. Section 2 briefly introduces the network model and our routing tree construction and maintenance strategy. Section 3 presents our event detection method and sensory data aggregation mechanism. Section 4 detects the event coverage and determines event sources. Section 5 presents the experimental evaluation of our technique. Section 6 reviews and discusses relevant techniques, and finally, Section 7 concludes this work.

## 2. Preliminaries: Routing Tree Construction and Maintenance

In underwater WSNs, a surface sonobuoy, serving as the sink node (*SN*), is deployed on the ocean surface accompanying radio and acoustic transceivers. Without loss of generality, only one sink node is deployed in the network, which is equipped with the Global Positioning System for determining its geographical location. Sensor nodes (denoted UV) are deployed in a three-dimensional Euclidean ocean space *D* ∈ ${ℜ}^{3}$, to perform collaborative monitoring tasks. Sensory data collected by UV are gathered and routed to *SN* for supporting certain applications [2]. Sensor nodes can be: (i) static, when attached with docks; (ii) semi-mobile, when deployed with buoys or ships; or (iii) mobile, when attached to autonomous underwater vehicles [26]. In this article, we assume that sensor nodes are semi-mobile, whose position changes continuously with the water dynamics. Besides, there should be anchor nodes and reference nodes deployed in the network, which are used for the localization of sensor nodes [2].

As presented in our previous work [22], based on the Channel-Aware Routing Protocol CARP [27], we have established a routing tree for gathering and routing sensory data to *SN* in a hop-by-hop fashion. Specifically, a HELLO control packet is flooded from SN throughout the whole network region during the network initialization phase:

(1) $$\begin{array}{c} \begin{array}{c} HELLO=<id_{src},HC> \end{array} \end{array}$$

where (i) $id_{src}$ is the unique identifier of a source sensor node uv∈ UV and (ii) *HC* is the hop count, which reflects the distance in hops for a certain sensor node uv to SN. The larger the value of *HC* is, the farther the distance is between uv and SN. The parent-child relation is established between neighboring sensor nodes, and a routing tree is constructed accordingly. Generally, the pitfalls of geographical routing protocols, including connectivity holes [28,29] and shallow zones [30], are avoided in our routing tree.

Sensor nodes in the underwater environment often drift along the water dynamics, which may induce the change of the network topology. This means that the parent-child relation may not hold and the routing tree should be adjusted. A heartbeat control packet HeartBt is adopted for the examination about whether the parent-child relation for uv and its parent sensor node $uv_{prt}$ is healthy or not:

(2) $$\begin{array}{c} \begin{array}{cc} HeartBt= & <id_{uv},id_{uv_{prt}},pid,crd_{uv},HC\left(uv\right),lq\left(uv,uv_{prt}\right)> \end{array} \end{array}$$

where (i) $id_{uv}$ (or $id_{uv_{prt}}$) represents the identifier of uv (or $uv_{prt}$); (ii) pid is the unique identifier of this *HeartBt* control packet; (iii) $crd_{uv}$ = ($crd_{uv}^{x}$, $crd_{uv}^{y}$, $crd_{uv}^{z}$) is the three-dimensional geographical coordinate for uv, which is identified through underwater localization techniques; and (iv) *lq*(uv, $uv_{prt}$) represents the link quality for uv and $uv_{prt}$.

When $uv_{prt}$ has received the *HeartBt* control packet from uv, which indicates that $uv_{prt}$ is still appropriate to serve as the parent node of uv, $uv_{prt}$ replies with an acknowledgment control packet ACK as the confirmation of this healthy situation:

(3) $$\begin{array}{c} \begin{array}{c} ACK=<id_{uv_{prt}},id_{uv},HC\left(uv_{prt}\right),pid,dist> \end{array} \end{array}$$

where dist represents the geographical distance between uv and $uv_{prt}$. Intuitively, dist is smaller than the communication radius *r* of sensor nodes, which is assumed to be the same for all sensor nodes in this article. Note that when dist is almost the same in value as *r*, the acoustic transmission loss should be high, the packet delivery ratio should be low and the bit error rate should be large [31,32]. This means that $uv_{prt}$ may not be appropriate to serve as the parent of uv, although $uv_{prt}$ is still within the communication radius of uv. Therefore, a robustness factor *ξ* ∈ (0, 1] is applied, such that $uv_{prt}$ is assumed appropriate to serve as the parent of uv, when dist is not larger than (*ξ* × *r*).

When no ACK is replied to by $uv_{prt}$, which suggests that $uv_{prt}$ cannot be the parent of uv any longer, a neighboring sensor node $uv_{y}$ should be chosen as the parent of uv through exchanging the EPING-PONGcontrol packets. We refer the reader to check our previous work [33] for this procedure. Specifically, uv will broadcast a EPING control packet:

(4) $$\begin{array}{c} \begin{array}{c} EPING=<id_{uv},id_{uv_{y}},g\left(uv_{y}\right),HC\left(uv\right),pid,L_{pkt}> \end{array} \end{array}$$

where $L_{pkt}$ = {<$pkt_{id_{src}}$, $pkt_{id}$>} represents a set of packet identifiers to be forwarded. $pkt_{id_{src}}$ specifies the sensor node that has generated this sensory data packet pkt and $pkt_{id}$ is the unique identifier of pkt. $uv_{y}$ replies with a PONG control packet to uv when it received this *EPING* control packet:

(5) $$\begin{array}{c} \begin{array}{c} PONG=<id_{uv_{y}},id_{uv},pid,HC\left(uv_{y}\right),queue,energy,lq\left(uv_{y},uv\right),bit\_mask_{L_{pkt}},bit\_mask_{JR}> \end{array} \end{array}$$

where *queue* specifies the available buffer space at $uv_{y}$ and *energy* refers to the resident energy at $uv_{y}$. $bit\_mask_{L_{pkt}}$ and $bit\_mask_{JR}$ are used for handling link asymmetries and interference. According to the set of PONG control packets received, uv selects the most appropriate sensor node, whose value of $g(uv_{y})$ is the largest, as its parent node.

Generally, a routing tree is responsible for routing data packets to *SN* along parent-child relations between sensor nodes, rather than selecting relay nodes whenever a sensor node has a data packet to be delivered. As shown by the evaluation result in [22], this strategy should reduce the energy consumption for sensory data gathering, aggregation and routing to *SN* to a certain extent, especially when the network topology is relatively steady.

## 3. Event Detection and Sensory Data Aggregation

Leveraging the routing tree constructed, we present our strategy for potential event identification and sensory data routing to *SN*. As presented in [34], due to the harsh environment that (underwater) WSNs monitor, faults or malicious attacks may occur, which may cause unreliability and inaccuracy of sensory readings. This indicates that an isolated and independent deviation of sensory data by a single sensor node from normal sensory data sensed by neighboring sensor nodes is highly possible in reflecting an outlier (or error). On the other hand, when several neighboring sensor nodes, whose sensory data show a deviation from normal sensory readings in a collective fashion, it is highly possible that an event has happened. Based on this observation, in this article, an event is defined as a particular phenomena where multiple neighboring sensor nodes exhibit a deviation in their sensory readings concurrently, while for a single sensor node, it is assumed as the occurrence of an outlier (or error). Consequently, sensor nodes, which may reflect the occurrence of events, should gather their sensory data and route to *SN* for event coverage detection. In this section, we propose a mechanism to gather and route sensory data to *SN*. Generally, sensory data of multiple neighboring sensor nodes are gathered by a sensor node (called a relay node) and are aggregated into a data packet for routing to *SN*.

When several neighboring sensor nodes detect a deviation of their sensory readings from a normal sensing range, an event may occur, and their sensory data should be gathered and routed to *SN* for event coverage detection and event source determination. A naive strategy is that each sensor node routes its sensory data to *SN* independently, leveraging the routing tree, which requires the forwarding of a relatively large number of data packets and, thus, is not energy efficient. Therefore, an appropriate relay node is to be selected for gathering and routing sensory data to *SN*.

As mentioned before, the *HeartBt* control packets are adopted for the maintenance of the routing tree. This control packet is extended for event detection and relay node selection in this technique as follows:

(6) $$\begin{array}{c} \begin{array}{c} HeartBt_{evt}=<id_{uv},id_{uv_{prt}},pid,crd_{uv},HC\left(uv\right),lq\left(uv,uv_{prt}\right),tag> \end{array} \end{array}$$

where a tag *tag* ∈ {0, 1} is appended for specifying whether sensory data of a certain sensor node deviated from a normal sensing range or not. Without loss of generality, 0 indicates the fact that sensory data are within the normal sensing range, while 1 indicates not.

When a sensor node $uv_{rel}$ receives a set of $HeartBt_{evt}$ control packets from its child sensor nodes $UV_{cld}$ and sibling sensor nodes $UV_{sib}$, $uv_{rel}$ will examine whether it can be a candidate relay node for some sensor nodes contained in $UV_{hb}$ = $UV_{cld}$ ∪ $UV_{sib}$. As presented by Algorithm 1, $uv_{rel}$ can be a candidate relay node for uv, when the following conditions can be satisfied:

- *HC*($uv_{rel}$) = *HC*(uv), for the case that uv ∈ $UV_{sib}$, or *HC*($uv_{rel}$) = (*HC*(uv) − 1), for the case that uv ∈ $UV_{cld}$. This means that $uv_{rel}$ is not farther from *SN* in hop count compared to uv. Therefore, the strategy that $uv_{rel}$ relays sensory data for uv may consume less, or no more in the worst case, energy than the strategy that uv relays sensory data for $uv_{rel}$.
- When *tag* for $uv_{rel}$ is 1 (Line 2), which means that sensory data for $uv_{rel}$ deviates from a normal sensing range and should be routed to *SN*. In this case, if there is another sensor node uv, whose sensory data deviates from a normal sensing range, as well, and should be relayed to *SN* by $uv_{rel}$, $uv_{rel}$ can be a candidate relay node for uv.
- When *tag* for $uv_{rel}$ is 0 (line 2), which means that sensory data for $uv_{rel}$ is within a normal sensing range. In this case, if there are no less than two sensor nodes whose sensory data deviate from a normal sensing range and should be relayed to *SN* by $uv_{rel}$, $uv_{rel}$ can be a candidate relay node for forwarding sensory data of these sensor nodes.

**Algorithm 1** Response for $HeartBt_{evt}$ control packets.**Require:**
-$uv_{rel}$ ∈ UV : a sensor node in the network-$UV_{hb}$ = $UV_{cld}$ ⋃ $UV_{sib}$ : a set of sensor nodes whose hop count is no smaller than that of $uv_{rel}$ and whose $HeartBt_{evt}$ control packets have been received by $uv_{rel}$
**Ensure:**
-$uv_{rel}$ may broadcast an EPONG control packet

1:cnt ← get the number of sensor nodes in $UV_{hb}$ whose *tag* is 12:**if** (cnt = 0) **or** (cnt = 1 **and** $uv_{rel}$(*tag*) = 0) **then**3: **return**4:**end if**5:$uv_{rel}$ broadcasts the control packet EPONG = <$uv_{rel}$, null, *pid*, *HC*($uv_{rel}$), *energy*, *queue*, *null*, $bit\_mask_{L_{pkt}}$, $bit\_mask_{JR}$, cnt>


It is worth mentioning that, given a sensor node uv whose sensory data deviate from a normal sensing range, when any sensor node $uv_{ngb}$, neighboring to uv, cannot satisfy the condition specified by Line 2 in Algorithm 1, uv corresponds to an outlier (or error), whose sensory data should not be routed to *SN*.

When $uv_{rel}$ is examined to be a candidate relay node for neighboring sensor nodes, it broadcasts an EPONG control packet for the competition of being the relay node:

(7) $$\begin{array}{c} \begin{array}{c} EPONG=<id_{uv},null,pid,HC\left(uv_{rel}\right),queue,energy,null,bit\_mask_{L_{pkt}},bit\_mask_{JR},cnt> \end{array} \end{array}$$

where the parameters for target sensor node and link quality are set to *null*. Different from the PONG control packet in the routing tree maintenance [22], the parameter cnt specifies the number of sensor nodes whose sensory data should be aggregated and forwarded by $uv_{rel}$. Generally, the larger the cnt is, the smaller the number of sensory data packets is to be routed to *SN*, although the relatively larger the sensory data packets are in size. This strategy should decrease the network traffic and reduce the energy consumption of the whole network to a certain extent.

As presented in Algorithm 2, when a sensor node uv receives several EPONG control packets, an appropriate sensor node should be selected as the relay node for uv. As presented in Line 3, when a candidate sensor node $EPONG_{cur}$.$uv_{rel}$ can:

- relay sensory data for a larger number of sensor nodes, which may reduce the number of sensory data packets to be forwarded in the network, or
- have more remaining energy with respect to its hop count (reflected by $EPONG_{cur}$.energy ÷ $EPONG_{cur}$.HC), which may promote the balance of energy consumption between sensor nodes and, thus, prolong the network lifetime.
  It is worth noting that when (i) the condition $EPONG_{cur}$.HC ≥ $EPONG_{sel}$.HC holds, which means that $EPONG_{cur}$.$uv_{rel}$ is not nearer *SN* in hop count in comparison with $EPONG_{sel}$.$uv_{rel}$, but (ii) the condition $EPONG_{cur}$.energy ÷ $EPONG_{cur}$.HC > $EPONG_{sel}$.energy ÷ $EPONG_{sel}$.HC holds, which means that $EPONG_{cur}$.$uv_{rel}$ can forward a larger number of sensory data packets than $EPONG_{sel}$.$uv_{rel}$, $EPONG_{cur}$.$uv_{rel}$ is assumed more appropriate than $EPONG_{cur}$.$uv_{rel}$ to serve as the relay node,

$EPONG_{cur}$.$uv_{rel}$ is chosen as the relay node for uv. Consequently, uv sends an acknowledge control packet ACK to $uv_{rel}$ for the confirmation of this selection (Line 8). Note that when $EPONG_{cur}$.$uv_{rel}$ is examined not as a candidate relay node for uv, $EPONG_{cur}$.$uv_{rel}$ ignores the received EPONG control packet, and no ACK control packet will be sent from uv to $uv_{rel}$.
**Algorithm 2** Relay node selection.**Require:**
-uv ∈ UV : a sensor node in the network-$EPONG_{set}$ : a set of EPONG control packets received by uv
**Ensure:**-$uv_{rel}$ : a sensor node to be selected for relaying sensory data of uv
1:$EPONG_{sel}$ ← ∅, where ∅ means an empty set2:**for** $EPONG_{cur}$ ∈ $EPONG_{set}$ **do**3: **if** ($EPONG_{cur}$.cnt > $EPONG_{sel}$.cnt) **or** (($EPONG_{cur}$.cnt = $EPONG_{sel}$.cnt) **and** ($EPONG_{cur}$.energy ÷ $EPONG_{cur}$.HC > $EPONG_{sel}$.energy ÷ $EPONG_{sel}$.HC)) **then**4:   $EPONG_{sel}$ ← $EPONG_{cur}$5: **end if**6:**end for**7:$uv_{rel}$.id ← $EPONG_{sel}$.$id_{uv}$8:uv sends an acknowledge control packet ACK to $uv_{rel}$


**Figure 1:**
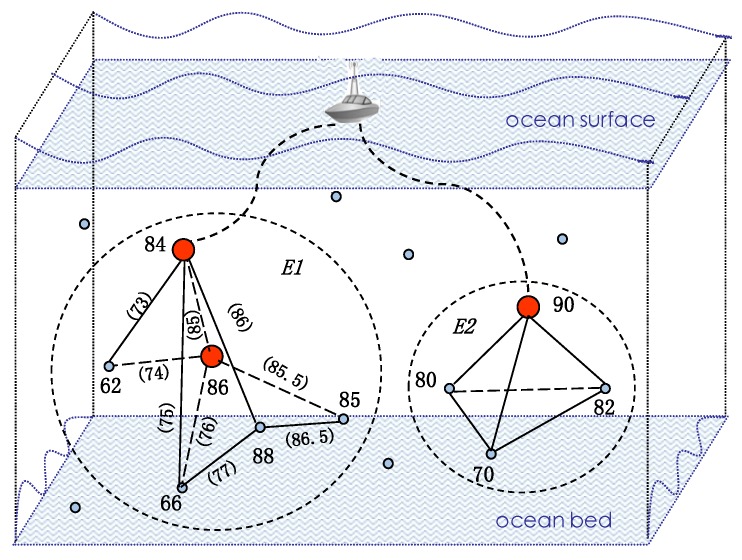
An example of event coverage and event sources. Specifically, the sensor node with the mark of 86 is the relay node for the sensor nodes with marks of 66, 88 and 85, and the sensor node with the mark of 84 is the relay node for the sensor nodes with marks of 62, 86 and itself. There are two events (*i.e*., *E1* and *E2*), corresponding to two connected components in this graph, and event sources are the sensor nodes with marks of 84 and 86 for *E1* and with the mark 90 for *E2*, respectively.

An example of the relay node selection is shown in Figure 1, where (i) the sensor node with a mark of 86 is the relay node for the sensor nodes with marks of 66, 88 and 85 and (ii) the sensor node with a mark of 84 is the relay node for the sensor nodes with marks of 62, 86 and itself.

This mechanism may make overload an optimal relay node $uv_{rel}$ responsible for a relatively large number of sensory data packets in a certain time slot, while other relay nodes $UV_{rel}$, which are not as optimal as $uv_{rel}$ in this time slot, may relay sensory data for quite a few sensor nodes. This may cause a relatively large amount of energy to be consumed for $uv_{rel}$ at this moment. Therefore, $uv_{rel}$ may be found not as an optimal relay node in the following time slots, and another sensor node in $UV_{rel}$ is found optimal instead. This means that different sensor nodes may serve as optimal rely nodes in different time slots, which may cause the energy consumption of sensor nodes to be almost the same as the average in the long term. We argue that this strategy can avoid the energy over-consumption of any single sensor node and, thus, prolong the network lifetime somehow.
**Algorithm 3** Sensory data gathering and aggregation.**Require:**
-$uv_{rel}$ : a relay sensor node-$UV_{evt}$ : a set of sensor nodes whose sensory data should be relayed by $uv_{rel}$
**Ensure:**
-$SRYDT_{uv}$ : a set of sensory data to be gathered and routed by $uv_{rel}$

1:$uv_{rel}$ sends a *getData* = <$id_{uv}$, {$\overset{-}{id_{uv}}$}, *pid*, *HC*($uv_{rel}$)> control packet to ∀ $uv_{evt}$ ∈ $UV_{evt}$2:$cnt_{sryDt}$ ← 03:**while** $uv_{rel}$ receives a $sryDt_{uv}$ data packet from a sensor node $uv_{evt}$ ∈ $UV_{evt}$ **do**4: $SRYDT_{uv}$ ← $SRYDT_{uv}$ ∪ {$sryDt_{uv}$}5: $cnt_{sryDt}$ ← $cnt_{sryDt}$ + 16: **if** $cnt_{sryDt}$ ≥ *sizeOf*($UV_{evt}$) **then**7:   **break**8: **end if**9:**end while**10:$uv_{rel}$ relays $SRYDT_{uv}$ to *SN*


As presented in Algorithm 3, when $uv_{rel}$ receives ACK control packets from a set of neighboring sensor nodes $UV_{evt}$, $uv_{rel}$ will send a *getData* control packet to all sensor nodes in $UV_{evt}$ only (Line 1):

(8) $$\begin{array}{c} \begin{array}{c} getData=<id_{uv},\left\{\overset{-}{id_{uv}}\right\},pid,HC\left(uv_{rel}\right)> \end{array} \end{array}$$

where $\overset{-}{id_{uv}}$ refers to the identifier of a sensor node $uv_{evt}$ ∈ $UV_{evt}$. $uv_{evt}$ will forward its sensory data to $uv_{rel}$ in terms of the format:

(9) $$\begin{array}{c} \begin{array}{c} sryDt_{uv}=<\overset{-}{id_{uv}},crd_{uv},val_{uv},atr_{uv}> \end{array} \end{array}$$

where $val_{uv}$ is the value of sensory data, and $atr_{uv}$ refers to the attribute to be sensed by $uv_{evt}$. Whenever $uv_{rel}$ receives the sensory data packet $sryDt_{uv}$ from any sensor node (Line 4), $uv_{rel}$ aggregates it into $SRYDT_{uv}$ (Line 4). When sensory data for all sensor nodes contained in $UV_{evt}$ have been gathered (Lines 5–8), $uv_{rel}$ routes the aggregated data packet to *SN* through the routing tree (Line 10). The function *sizeOf* () in Line 6 returns the number of elements contained in a certain set.

## 4. Event Coverage Detection and Event Sources Determination

When *SN* receives sensory data $sryDt_{uv}$ ∈ SRYDT from all sensor nodes, whose sensory data may exhibit a deviation from a normal sensing range, the event coverage is to be detected as introduced by Algorithm 4. A weighted graph $g_{evt}$ = ($UV_{evt}$, $EG_{evt}$, $WGT_{evt}$) is adopted to represent the event coverage, where $UV_{evt}$ and $EG_{evt}$ represent sensor nodes and edges connecting these sensor nodes in this graph, while $WGT_{evt}$ is the weight specified upon the edges $EG_{evt}$.
**Algorithm 4** Event coverage detection.**Require:**
-SRYDT : a set of sensory data received by *SN*
**Ensure:**
-$g_{evt}$ = ($UV_{evt}$, $EG_{evt}$, $WGT_{evt}$) : a weighted graph representing the event coverage

1:**for each** $sryDt_{uv1}$ ∈ SRYDT **do**2: **for each** $sryDt_{uv2}$ ∈ SRYDT - {$sryDt_{uv1}$} **do**3:   **if** ∥$sryDt_{uv1}$.$crd_{uv1}$ - $sryDt_{uv2}$.$crd_{uv2}$ ∥ > *r* **then**4:      **continue**5:   **end if**6:   $eg_{evt}$ ← make an edge connecting $sryDt_{uv1}$.$id_{uv1}$ and $sryDt_{uv2}$.$id_{uv2}$7:   **if** $eg_{evt}$ ∈ $g_{evt}$.$EG_{evt}$ **then**8:      **continue**9:   **end if**10:   $g_{evt}$.$UV_{evt}$ ← $g_{evt}$.$UV_{evt}$ ∪ {$sryDt_{uv1}$.$id_{uv1}$, $sryDt_{uv2}$.$id_{uv2}$}11:   $g_{evt}$.$EG_{evt}$ ← $g_{evt}$.$EG_{evt}$ ∪ {$eg_{evt}$}12:   wgt($eg_{evt}$) ← ($sryDt_{uv1}$.$val_{uv}$ + $sryDt_{uv2}$.$val_{uv}$) ÷ 213:   $g_{evt}$.$WGT_{evt}$ ← $g_{evt}$.$WGT_{evt}$ ∪ {wgt($eg_{evt}$)}14: **end for**15:**end for**


Given two sensor nodes $sryDt_{uv1}$.$id_{uv1}$ and $sryDt_{uv2}$.$id_{uv2}$, if the Euclidean distance between them is larger than the communication radius of sensor nodes (denoted by the symbol *r* in Line 3), no edge can connect these two sensor nodes (Lines 3–5). The symbol ∥.∥ means the computation of the Euclidean distance. When an edge $eg_{evt}$ (Line 6) connecting these two sensor nodes has been studied, another pair of sensor nodes are to be explored afterwards (Lines 7–9). Otherwise, these two sensor nodes are inserted into $g_{evt}$.$UV_{evt}$ (Line 10), and $eg_{evt}$ is inserted into $g_{evt}$.$EG_{evt}$ (Line 11), accordingly. The weight specified upon $eg_{evt}$ is computed as the average of sensory data value by these two sensor nodes (Lines 12–13). Consequently, a weighted graph (*i.e*., $g_{evt}$) that represents the event coverage has been established. The coverage of potential event(s) corresponds to the merger of the spherical space, which is prescribed by the location of each sensor node (denoted $sryDt_{uv}$.$id_{uv}$) and the communication radius *r* of $sryDt_{uv}$.$id_{uv}$.

It is worth noting that $g_{evt}$ may comprise several smaller connected components, and each connected component corresponds to the coverage of a single event. This is due to the fact that several events may occur concurrently at different geographical sub-regions in the network in a certain time slot, and geographical gaps may exist between the coverage of these events.

An example of event coverage is shown in Figure 1, where two events (denoted as *E1* and *E2*) are detected, for sensor nodes with marks of (84, 62, 86, 85, 66, 88)and (90, 80, 82, 70), respectively. They correspond to the two connected components in this graph. The coverage of an event (for instance, *E1*) is the merger of the spheres in which sensor nodes are contained (for instance, the sensor nodes with marks of 84, 62, 86, 85, 66 and 88 for *E1*).

**Algorithm 5** Event source determination.**Require:**
-$g_{evt}$ = ($UV_{evt}$, $EG_{evt}$, $WGT_{evt}$) : a weighted graph representing the event coverage
**Ensure:**
-$UV_{src}$ : a set of top *k*% sensor nodes representing event sources

1:$cnt_{src}$ ← *round*(*k*% × *sizeOf*($g_{evt}$.$UV_{evt}$))2:**for each** $uv_{src}$ ∈ $g_{evt}$.$UV_{evt}$ **do**3: $EG_{src}$ ← retrieve a set of edges from $g_{evt}$.$EG_{evt}$, such that each edge connects $uv_{src}$4: **for each** $eg_{src}$ ∈ $EG_{src}$ **do**5:   $wgt_{src}$ ← retrieve the weight for $eg_{src}$ from $g_{evt}$.$WGT_{evt}$6:   $wgt_{sum}$ ← $wgt_{sum}$ + $wgt_{src}$7: **end for**8: **if** *sizeOf* ($UV_{src}$) <$cnt_{src}$ **then**9:   $UV_{src}$ ← $UV_{src}$ ∪ {$uv_{src}$}10: **else**11:   $uv_{lst}$ ← retrieve a sensor node from $UV_{src}$ whose $wgt_{sum}$ is the smallest12:   **if** $uv_{lst}$.$wgt_{sum}$<$uv_{src}$.$wgt_{sum}$ **then**13:      $UV_{src}$ ← $UV_{src}$ ∪ {$uv_{src}$} - {$uv_{lst}$}14:   **end if**15: **end if**16:**end for**


After the detection of event coverage, the sources of events need to be determined, which usually correspond to the pivots for responses to be taken. Intuitively, event sources reflect sensor nodes whose sensory data deviate the most in value from the normal sensing range. Without loss of generality, the barycenters [25] are identified in $g_{evt}$ to represent event sources. A barycenter in a weighted graph refers to a node in this graph, such that the sum of the weights specified upon the edges connecting this node is the largest. Generally, around *k*% sensor nodes in $g_{evt}$.$UV_{evt}$ are selected as event sources. Note that the function *round*() in Line 1 of Algorithm 5 is the rounding function for decimals, and the function *sizeOf* () returns the number of elements contained in a certain set.

Given a sensor node $uv_{src}$ contained in $g_{evt}$.$UV_{evt}$ (Line 2), the edges $EG_{src}$ connecting $uv_{src}$ are retrieved (Line 3), and the sum of weights specified upon $EG_{src}$ is calculated (Lines 4–7). $uv_{src}$ can be a potential event source when one of the following conditions can be satisfied:

- there exists slots for candidate event sources (Lines 8–9) or
- there exists another candidate event source $uv_{lst}$ (Line 11), which is not as appropriate as $uv_{src}$ (Line 12). This is specified by the condition of $uv_{lst}$.$wgt_{sum}$ < $uv_{src}$.$wgt_{sum}$. Consequently, $uv_{lst}$ is replaced by $uv_{src}$ in $UV_{src}$ (Line 13).

An example is shown in Figure 1, where two event sources for *E1* are the sensor nodes with marks of 84 and 86, and one event source for *E2* is the sensor node with the mark of 90.

It is worth noting that some connected components in $g_{evt}$ may contain the majority of event sources, since sensory data for sensor nodes in these connected components may deviate to a relatively large extent from the normal sensing range; while the situation for sensor nodes contained in the other connected components is not that serious somehow. When this situation is encountered, it is appropriate that prompt and proper responses should be taken to remedy those more serious event sources.

## 5. Implementation and Evaluation

The prototype has been implemented in a Java program, and experiments are conducted for evaluating the performance and efficiency of our event coverage detection and event source determination mechanisms. In the following, we introduce the environment settings, present the results of experiments and compare our technique to CARPas the protocol for routing sensory data to *SN*.

### 5.1. Environment Settings

The parameter settings for our experiments are presented in Table 1. Specifically, the network is deployed in a three-dimensional underwater space with the geographical volume of 1 × 2 × 0.5 km${}^{3}$. The number of sensor nodes is set to 61, where one sink node (*SN*) is deployed as the surface sonobuoy, while 60 sensor nodes are deployed in the underwater environment with different depths ranging from 0.01 km to 0.5 km. The transmission radius of sensor nodes (*r*) is set to 0.15 km, and it can be changed to other values for diverse experimental purposes, which makes the connection of sensor nodes with the packet delivery from any sensor node to *SN* be within four hops. The size of an EPING, EPONG, HELLO, $HeartBt_{evt}$, *getData* and ACK control packet is set to *11B*, *7B*, *7B*, *11B*, *6B* or *6B*, respectively. The robustness factor for the parent-child relation determination is set to 0.7, which means that the geographical distance between parent and child sensor nodes should be no larger than (0.7 × *r*) = 0.105 km. The smoothing factor adopted for computing the link quality is set to 0.7. The energy consumption for transmitting a data or control packet is set to 2.8 W or 1.5 W, respectively. Generally, one or more events are generated at each time slot, and sensory data for potential events are gathered and routed to *SN* through the routing tree. Experiments are conducted over 10, 20 or 30 time slots for evaluation purposes, and these experiments are performed on a desktop with an Intel(R) Core(TM) i5-3470 CPU @ 3.20 GHz, an 8-GB memory and the 64-bit Windows system.

### 5.2. Experimental Evaluation

Experiments have been conducted for evaluating the performance and efficiency of our event coverage detection and sensory data routing mechanisms. Without loss of generality, the network space is defined as a rectangular region, and *SN* is located at the center of the ocean surface with the geographical coordinate of (0.5, 1, 0), where the *z*-coordinate corresponds to the depth of *SN* (or sensor nodes). The results of our experimental evaluation are presented and discussed in the following.

**Table 1:** Experiential parameters settings.

Parameter Name	Value
Simulation network region (km${}^{3}$)	1 × 2 × 0.5
Number of sensor nodes (including one sink node)	61
Transmission radius *r* (km)	0.15
Time slots for experiments	10, 20, 30
EPING or $HeartBt_{evt}$ control packet size (*B*)	11
EPONG or HELLO control packet size (*B*)	7
ACK and *getData* control packet size (*B*)	6
Data packet payload size (*B*)	100
Robustness factor for the parent-child relation determination	0.7
Smoothing factor for the link quality computation	0.7
Power for transmitting a data packet (*W*)	2.8
Power for transmitting a control packet (*W*)	1.5

**Figure 2:**
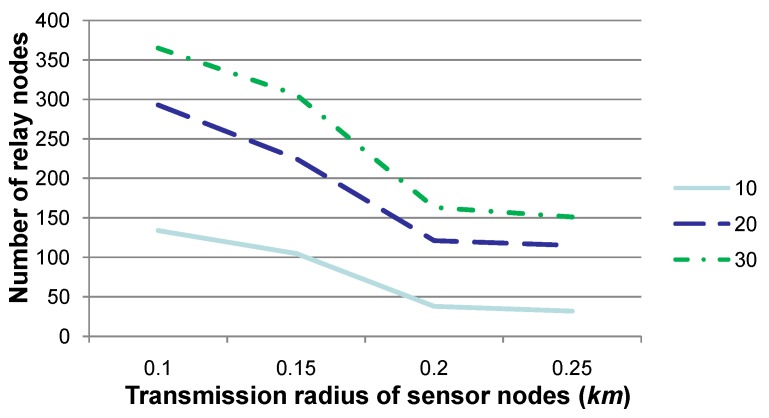
Comparison of the number of relay nodes when the transmission radius *r* is set to various values, where 10, 20 and 30 specify the number of time slots for our experiments. This figure shows that the number of relay nodes declines when the transmission radius is set to a relatively large value.

**Figure 3:**
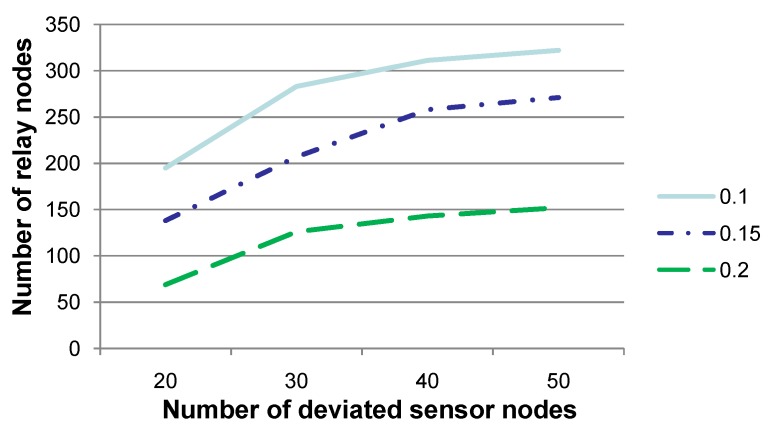
Comparison of the number of relay nodes when the number of sensor nodes (denoted cnt), whose sensory data deviate from a normal sensing range, is set to various values. This figure shows that the number of relay nodes increases significantly when cnt is set to a relatively large value.

**Figure 4:**
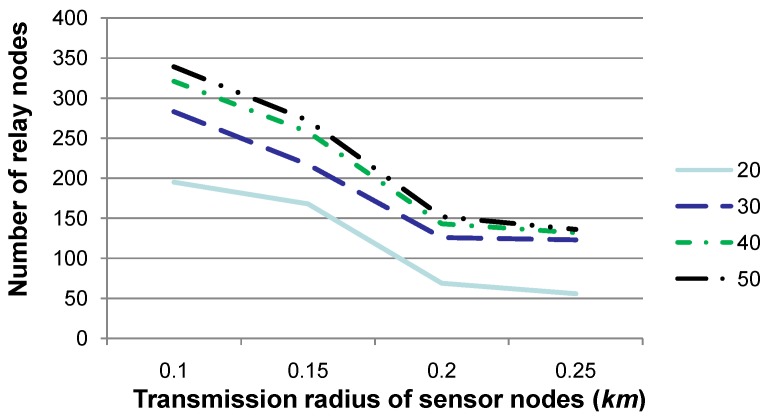
Comparison of the number of relay nodes when the transmission radius *r* is set to various values and the number of deviated sensor nodes is set to 20, 30, 40 or 50, respectively. This figure shows that the number of relay nodes is mostly impacted by the density of deviated sensor nodes, which is determined by *r* and the number of deviated sensor nodes in the network region.

Figure 2 shows the number of relay nodes when the transmission radius *r* is set to 0.1, 0.15, 0.2 or 0.25, respectively. The experiments are conducted over 10, 20 or 30 contiguous time slots, and the number of sensor nodes, whose sensory data may deviate from a normal sensing range, is set as 30 at each time slot. Note that “number of relay nodes” in this figure, as well as that in Figure 3 is the number in total for all relay nodes in these 10, 20 or 30 time slots. Due to the water dynamics, it is assumed that no more than five sensor nodes may drift away during each time slot, and their *x*- and *y*-coordinates may change no more than 3–5 m, while their *z*-coordinate may change no more than 0–3 m. Figure 2 shows that the number of relay nodes declines to a certain extent when *r* is set to a relatively large value, since the transmission region of a sensor node is relatively larger, and hence, more sensor nodes may select the same relay node according to Algorithm 2 for sensory data gathering and routing to *SN*. This figure also shows that the number of relay nodes is non-linear with the value of the transmission radius *r*, since the transmission region (*i.e*., $\frac{4}{3}\times \pi \times {\left(r_{qry}\right)}^{3}$) increases much quicker than *r*. Consequently, when *r* increases, the number of sensor nodes within the transmission region of a certain relay node may increase to a large extent, which results in a decrease in the number of relay nodes. Besides, the number of relay nodes shown in this figure is almost the same when *r* is set to 0.2 or 0.25. After examining the deployment of sensor nodes, it is found that, given two candidate relay nodes, the increase for the number of sensor nodes that one candidate node can relay is almost the same as that for another. This means that the determination of relay nodes may not change for sensor nodes, and hence, the number of relay nodes may not change, as well. This fact indicates that the density of deviated sensor nodes (a more detailed discussion is presented in Figure 4), which is determined by the number of deviated sensor nodes and the communication radius, is the key factor for determining the number of relay nodes.

Figure 3 shows the number of relay nodes when the number of sensor nodes (denoted cnt), whose sensory data deviate from a normal sensing range, is set to 20, 30, 40 or 50, respectively. The communication radius *r* is set to 0.1, 0.15 or 0.2, respectively. Note that the experiments for *r* as 0.25 are not discussed, since *r* as 0.25 is relatively too large with respect to the network region, and the results of the experiments may not be convincing. This figure shows that the number of relay nodes increases to an extent when cnt is set to a relatively large value. Note that when cnt is quite large (e.g., 40 or 50), the increasing of the number of relay nodes is relatively small. This is due to the fact that when cnt is relatively large, sensor nodes, whose sensory data deviate from a normal sensory range, are densely distributed in the network. Consequently, a relay node may have to relay sensory data for a relatively larger number of deviated sensor nodes, but relay nodes may not need to be newly added. In this setting, the workload of relay nodes should be increased, which should cause the increase of energy consumption in total. However, the number of relay nodes may not increase to an extent.

Figure 4 shows the number of relay nodes when the transmission radius *r* is set to 0.1, 0.15, 0.2 or 0.25, respectively, while the number of deviated sensor nodes is set to 20, 30, 40 or 50, respectively. The other parameters are set to the same values as those in Figure 3. Figure 4 shows that the increase of the number of relay nodes is non-linear with that for the number of deviated sensor nodes. In fact, the density of deviated sensor nodes in the network region is the key factor for determining the number of relay nodes, where the density can be represented as the average number of deviated sensor nodes contained in a sphere whose radius is *r*. Generally, when deviated sensor nodes are relatively sparely deployed in the network region (for instance, the number of deviated sensor nodes is 20 or 30), newly-added deviated sensor nodes $UV_{dev}$ may require newly-added relay nodes for sensory data gathering and routing to *SN*, since existing relay nodes may hardly cover $UV_{dev}$. On the other hand, when the number of deviated sensor nodes is large enough (for instance, 40 or 50), relay nodes may have covered the whole network region already. Consequently, newly-added deviated sensor nodes can be relayed by existing relay nodes, although their workload is much heavier than before. As argued in Figure 2, the number of relay nodes is mostly decided by the density, rather than the number of deviated sensor nodes.

**Figure 5:**
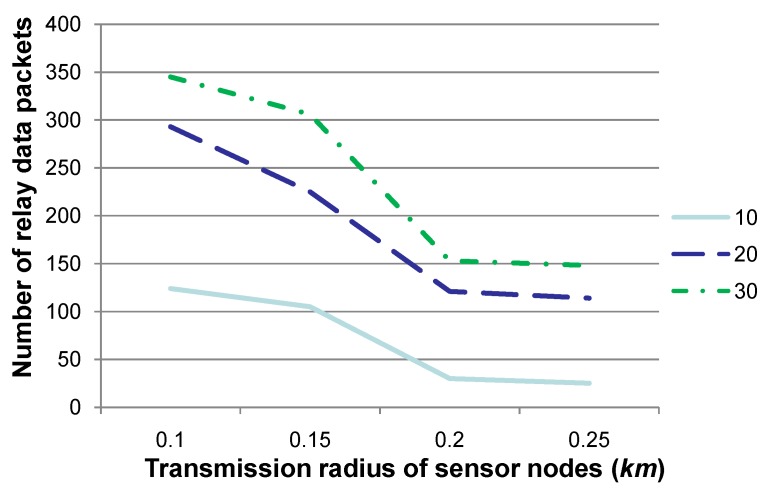
Comparison of the number of relay data packets when the transmission radius *r* is set to various values. This figure shows that the number of relay data packets declines when the transmission radius is set to a relatively large value.

Figure 5 shows the number of relay data packets when the transmission radius *r* is set to 0.1, 0.15, 0.2 or 0.25, respectively. Similar to “number of relay nodes” in Figure 2, “number of relay data packets” in this figure specifies the number in total for all relay nodes in these 10, 20 or 30 time slots. The other parameters are set to the same values as those in Figure 2. Figure 5 shows that the number of relay data packets decreases when *r* is set to a relatively large value. However, when *r* is large enough (e.g., 0.2 or 0.25), the number of relay data packets is almost the same. Similar to the explanation in Figure 2, the relation for sensor nodes and the corresponding relay nodes may not change somehow when *r* is large. This means that the number of relay data packets may not change, as well, although hop counts may be decreased when routing relay data packets to *SN*, which may cause the decrease of energy consumption.

**Figure 6:**
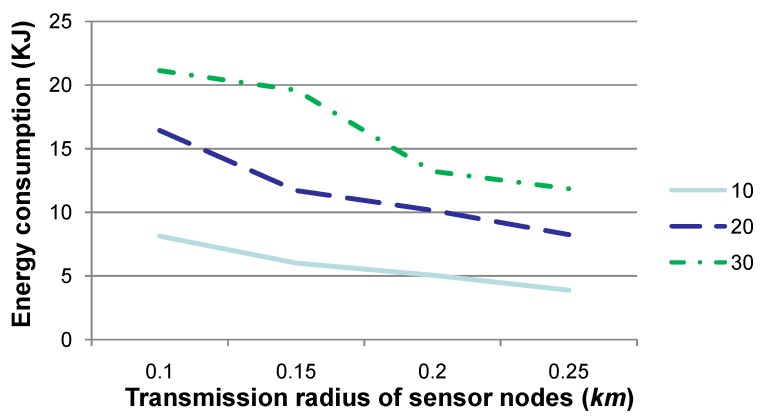
Comparison of energy consumption when the transmission radius *r* is set to various values. Generally, the larger the value of *r* is, the less the energy consumption is for sensory data gathering and routing to *SN*.

Figure 6 shows the energy consumption when the transmission radius *r* is set to 0.1, 0.15, 0.2 or 0.25, respectively. The other parameters are the same as those in Figure 2. Similar to “number of relay nodes” in Figure 2, “energy consumption (KJ)” in Figure 6 specifies the energy consumed in total for all relay nodes in these 10, 20 or 30 time slots. Figure 6 shows that the energy consumption decreases when *r* is set to a relatively large value. This is due to the fact that the number of relay data packets decreases when *r* increases. As indicated by the values in Table 1, the smaller the number of data (or control) packets is to be transmitted, the less the energy is to be consumed. As presented by Figure 5, when *r* is large enough, the number of relay data packets is almost steady. Therefore, the energy consumption may not decrease to an extent. It is worth mentioning that when *r* is large, there may exist relay nodes that may relay sensory data for a single sensor node. This may induce an imbalance of energy consumption between relay sensor nodes and may be harmful to the network lifetime. Consequently, a tradeoff should be considered when setting a value for *r* in real applications.

**Figure 7:**
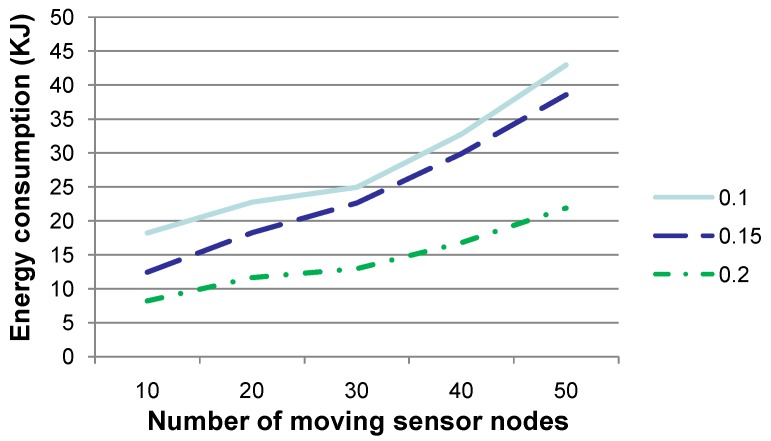
Comparison of energy consumption when the number of sensor nodes, which drift with the water dynamics and change their coordinates, is set to various values. This figure shows that the larger the number of sensor nodes changing their coordinates, the more energy is to be consumed.

Figure 7 shows the energy consumption when the number of sensor nodes, which drift with the water dynamics and change their coordinates, is set to 10, 20, 30, 40 or 50, respectively, where the *x*- and *y*-coordinates of these sensor nodes change no more than 3–5 m and their *z*-coordinate changes no more than 0–3 m, per time slot. The energy consumption is the amount in total for the experiments conducted for 20 time slots. The other parameters are set to the same values as those in Figure 2. As presented by Algorithm 2, the larger the number of sensor nodes whose coordinates change is, the larger the number of relay nodes that have to be re-selected and the larger the number of $HeartBt_{evt}$-EPONG control packets to be transmitted. Therefore, more energy is to be consumed during the relay nodere-selection phase, as shown in Figure 7. This figure indicates that our technique is more energy efficient when the network topology is relatively steady.

**Figure 8:**
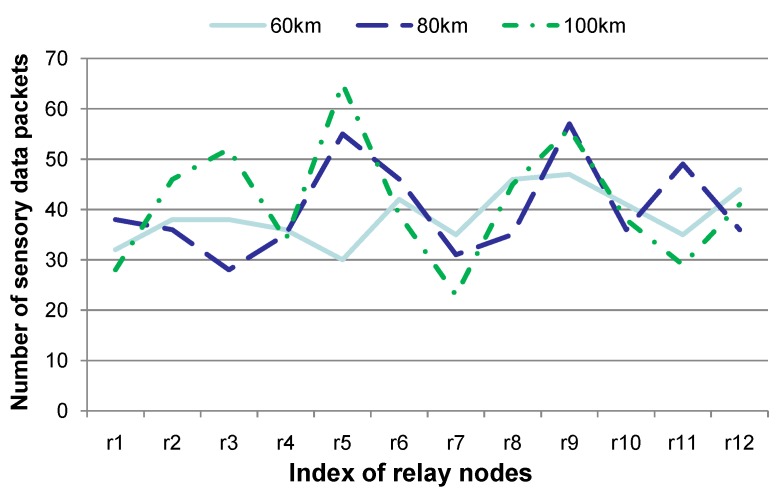
Comparison of the number of sensory data packets when sensor nodes are deployed in diverse skewness distributions in the network region. This figure shows that different sensor node deployment strategies may have a relatively large impact on the number of sensory data packets to be forwarded by certain relay nodes.

Figure 8 shows the number of sensory data packets that a certain relay node delivers, when sensor nodes are deployed in the network space under various skewness distributions. Intuitively, two sensor nodes (denoted $uv_{1}$ and $uv_{2}$) can have a link, when the geographical distance dst between $uv_{1}$ and $uv_{2}$ is shorter than the communication radius *r*. Given the set dst}, the variance is adopted to represent the skewness degree of the sensor node distribution. Generally, when the variance is smaller, which means that the geographical distances between sensor nodes are more similar, sensor nodes are distributed more evenly in the network region. In our experiments, three kinds of sensor node distributions have been generated and their variances are 60 km, 80 km or 100 km, respectively. The experiments are conducted for 20 contiguous time slots, and the other parameters are set to the same values as those in Figure 5. Twelve relay nodes are selected for studying the number of sensory data packets to be forwarded, and these 12 relay nodes are represented as r1, ⋯, r12 in Figure 8. Note that these 12 relay nodes include those having the largest, and the smallest, number of sensory data packets. Figure 8 shows that the distribution of the number of sensory data packets is relatively even when the variance is relatively smaller (*i.e*., the variance is 60 km), although the number of sensory data packets for certain relay nodes may be smaller when the variance is relatively larger (r7, for instance). This is due to the fact that sensor nodes are distributed in a more skewed fashion when the variance is relatively larger. Therefore, the workload of relay nodes is more uneven, which is reflected by the number of sensory data packets in Figure 8. Note that relay nodes with a larger number of sensory data packets should consume more energy and may die earlier than the others. This is harmful to the network lifetime. Therefore, a relatively even distribution of sensor nodes is beneficial for balancing the energy consumption of relay nodes.

**Figure 9:**
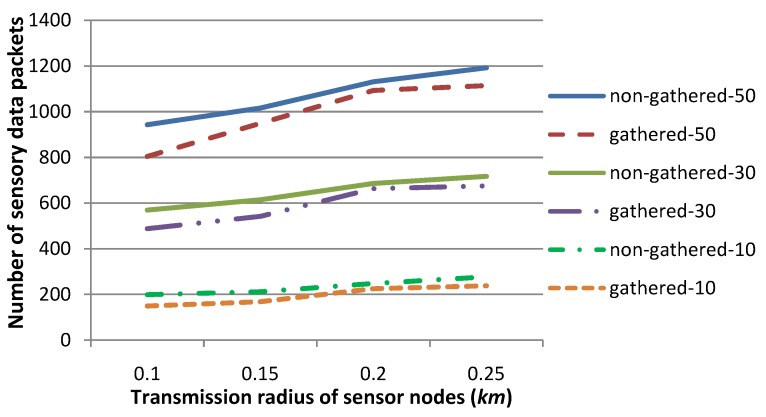
Comparison of the number of sensory data packets when the transmission radius *r* is set to various values, for the strategies that sensory data are gathered, or not gathered, by relay nodes. This figure shows that our gathered strategy requires a smaller number of sensory data packets than the number that non-gathered strategy requires, especially when *r* is set to a relatively small value.

**Figure 10:**
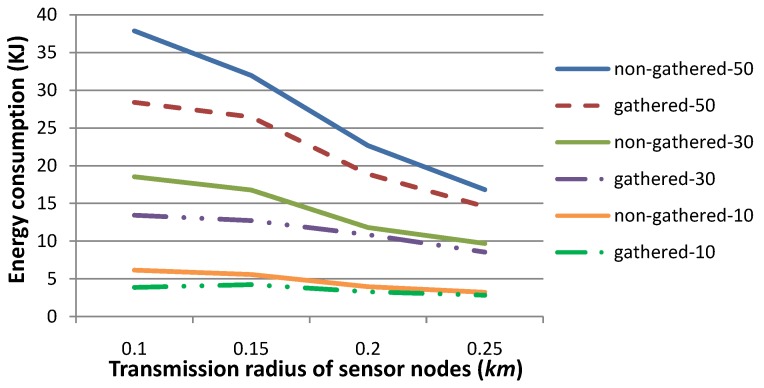
Comparison of the energy consumption when the transmission radius *r* is set to various values, for the strategies that sensory data are gathered, or not gathered, by relay nodes. This figure shows that the energy consumption for our gathered strategy is smaller than that for the traditional non-gathered strategy, especially when *r* is relatively small.

Figure 9 shows the number of sensory data packets, when sensory data are gathered by relay nodes, or are not gathered, and will be routed by individual sensor nodes through the routing tree independently. The transmission radius *r* is set to 0.1, 0.15, 0.2 or 0.25, respectively. The experiments are conducted for 20 contiguous time slots. There are 10, 30 or 50 sensor nodes in each time slot whose sensory data deviate from a normal sensing range, which are specified in Figure 9 and in Figure 10, as gathered, 10/30/50, and non-gathered, 10/30/50, respectively. The other parameters are set to the same values as those in Figure 5. Figure 9 shows that the number of sensory data packets is smaller for the gathered cases, when *r* is relatively smaller. This is due to the fact that sensory data of several sensor nodes can be gathered as a single data packet, which should be routed to *SN* with relatively large hops through the routing tree. Hence, the number of sensory data packets can be reduced to a large extent, in comparison with that of the non-gathered cases. This experiment shows the advantage of our sensory data gathering strategy than the traditional non-gathered one on reducing the number of sensory data packets.

Figure 10 shows the energy consumption for the gathered or non-gathered scenarios. As presented in Figure 9, sensory data packets are much fewer for gathered cases when the transmission radius *r* is set to a relatively smaller value, and hence, energy consumption is also quite less in this situation, although sensory data packets should be larger in size for the gathered strategy than for the non-gathered strategy. Consequently, our gathered strategy is more energy efficient, especially when *r* is set to a relatively small value.

### 5.3. Comparison with CARP for the Number of Control Packets and Energy Consumption

This section presents the result of our experiments for our sensory data gathering technique (denoted SDA in the following) with respect to CARP [27], where CARP serves as the protocol for routing sensory data to *SN*. As mentioned before, the routing strategy adopted in our technique is developed through improving the mechanism of CARP.

**Figure 11:**
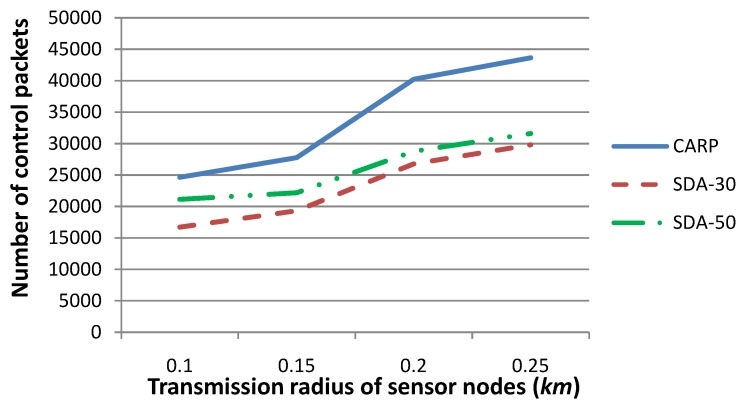
Comparison of the number of control packets for our technique (SDA) with respect to CARPas the routing protocol, when the transmission radius *r* is set to various values, and the number of sensor nodes, which drift with the water dynamics and change their coordinates, is set to various values. This figure shows that the number of control packets generated by our technique is much smaller than that of CARP, especially when the network topology is relatively steady.

Figure 11 shows the number of control packets generated by (i) our technique and (ii) CARP as the routing protocol, when the transmission radius is set to 0.1, 0.15, 0.2 or 0.25, respectively. The number of sensor nodes, which drift with the water dynamics and change their coordinates, is set to 30 or 50, respectively, where the *x*- and *y*-coordinates of these sensor nodes change no more than 3–5 m and their *z*-coordinate changes no more than 0–3 m, per time slot. There are 30 sensor nodes at each time slot whose sensory data deviate from a normal sensing range. The other parameters are set to the same values as those in Figure 5. Figure 11 shows that the number of control packets for CARP is the same when the number of moving sensor nodes varies, since CARP reselects relay nodes whenever sensory data are required to be routed to *SN*, and hence, the number of control packets is not impacted by the number of moving sensor nodes. On the other hand, the number of control packets generated by our technique is much smaller than that by CARP, when the number of sensor nodes, which drift with the water dynamics and change their coordinates, is relatively smaller. This is due to the fact that when the network topology is relatively steady, the number of parent nodes, which are determined in previous time slot(s) and can be reused for routing sensory data to *SN* in the forthcoming time slots, are relatively larger in number. Therefore, the number of $HeartBt_{evt}$-EPONG control packets is smaller. Besides, when the communication radius *r* is relatively large, the parent-child relation for a larger number of sensor nodes can be maintained, although their coordinates have been changed. This is reflected by Figure 11: the difference in the number of control packets is smaller for *r* = 0.25 than that for *r* = 0.1. Generally, a smaller number of control packets is generated by our technique than by CARP, especially when the network topology is relatively steady.

**Figure 12:**
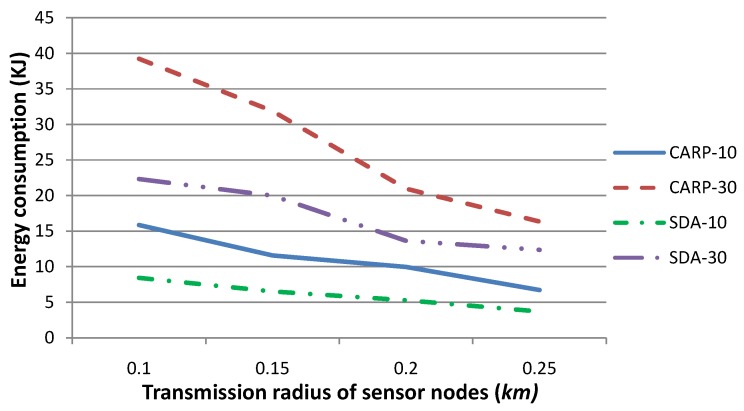
Comparison of energy consumption for our technique (SDA) with respect to CARP as the routing protocol, when the number of time slots is set to 10 and 30, respectively. This figure shows that the energy consumption for our technique is less than that of CARP, especially when the communication radius *r* is set to a relatively small value.

Figure 12 shows the energy consumption for (i) our technique and (ii) CARP as the routing protocol. The transmission radius *r* is set to 0.1, 0.15, 0.2 or 0.25, respectively. The experiments are conducted for 10 and 30 contiguous time slots. There are 30 sensor nodes at each time slot whose sensory data deviate from a normal sensing range. The other parameters are set to the same values as those in Figure 5. Figure 12 shows that the energy consumption of our sensory data aggregation strategy is less than that of CARP. This is due to the fact that sensory data, whose value has been varied significantly, are gathered and routed to *SN*. This means that the partial, rather than the whole of, sensory data should be routed to *SN* in a certain time slot. However in CARP, all sensory data should be routed to *SN*, and each data packet should be routed independently. Therefore, more energy is to be consumed for data gathering and routing. Figure 12 also shows that the difference of energy consumption becomes smaller when the transmission radius is set to a relatively large value, since a smaller number of hops are required when routing data packets to *SN*. Besides, our technique requires the maintenance of a routing tree, which induces some energy consumption. Generally, our technique is more energy efficient, especially when the transmission radius is a relatively small value.

**Figure 13:**
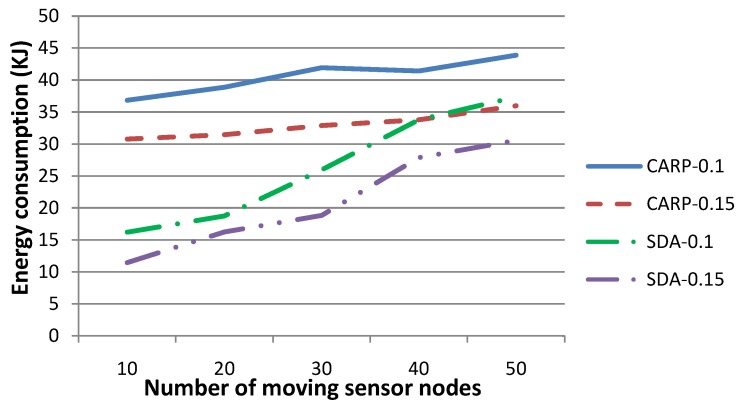
Comparison of energy consumption for our technique (SDA) with respect to CARP as the routing protocol, when the transmission radius *r* is set to 0.1 and 0.15, respectively, and the number of sensor nodes, which drift with the water dynamics and change their coordinates, is set to various values. This figure shows that the energy consumption for our gathered strategy is much smaller than that of CARP, especially when the number of moving sensor nodes is relatively small.

Figure 13 shows the energy consumption for (i) our technique and (ii) CARP as the routing protocol. The transmission radius *r* is set to 0.1 and 0.15, respectively. The number of sensor nodes, which drift with the water dynamics and change their coordinates, is set to 10, 20, 30, 40 or 50, respectively, where the *x*- and *y*-coordinates of these sensor nodes change no more than 3–5 m and their *z*-coordinate changes no more than 0–3 m, per time slot. The other parameters are the same as those in Figure 12. Figure 13 shows that our technique requires consuming more energy when the number of moving sensor nodes increases, while that for CARP is relatively steady (although large relatively). In fact, our technique may require one to re-select the parent nodes for sensory data gathering and routing to *SN*, while it can hardly reuse the parents determined in previous time slot(s), when a larger number of sensor nodes changes their coordinates frequently. Generally, the larger the number of parent nodes is to be reselected, the larger the number of $HeartBt_{evt}$-EPONG control packets to be transmitted and the more the energy is to be consumed during the sensory data routing procedure. Figure 13 shows that the energy consumption increases quickly along the increase of the number of moving sensor nodes. On the other hand, CARP requires one to select the parent nodes whenever a data packet is to be routed. This strategy suggests that the number of moving sensor nodes may not impact the energy consumption to an extent. Consequently, our technique is impacted by the number of moving sensor nodes and is more energy efficient, especially when the network topology is relatively steady.

## 6. Related Work and Comparison

Underwater WSNs are becoming a more pressing research topic, due to the rapid development of sensing technologies and the urgent requirement for studying the vast under-explored volume of ocean. Since the underwater environment is harsh and the communication cost is high, energy efficiency is a factor of core importance when detecting events in underwater WSNs [8]. Traditional techniques have explored the event detection and coverage determination in the underwater environment. In [17], the authors proposed to determine event locations through a sensor visualization approach. Intuitively, there may be multiple events occurring concurrently; however, these events may be located relatively sparsely in a relatively large network space. Since the water current is usually small in velocity in most situations, sensor nodes can be localized accurately. The drawback is the fact that this may cause relatively large energy consumption, especially when few events occur and quite a few sensor nodes are not involved in these events. To mitigate this problem, the concept of virtual sensors is introduced, which corresponds to an aggregation point for multiple physical sensors. Specifically, sensor nodes are assigned into several clusters, and head nodes in clusters delegate and function as virtual sensors for gathering and routing sensory data to sink node(s). Consequently, event detection and localization is achieved with the sensory data of virtual sensors. The compressive sensing technique is adopted for recovering the signal with insufficient measurements in a sparse environment, and this strategy can be adopted to improve our approach when sensor nodes are sparsely distributed. Due to the water dynamics, sensor nodes may drift away, and clusters, which are generated in previous time slots, may change their topology afterwards. The adjustment of cluster structures may be energy consuming. Virtual nodes (*i.e.*, cluster head nodes) may have to be re-selected, and thus, the topology for sink node(s) and virtual nodes may be re-established after certain time slots. On the contrary, our approach depends on the routing tree for gathering and routing sensory data, where hop counts are adopted for the avoidance of pitfalls, including connectivity holes and shallow zones. The maintenance of the routing tree is mostly to examine parent-child relations between sensor nodes, which are local and not energy consuming.

Due to the huge volume of ocean space, autonomous underwater vehicles (AUVs) are usually deployed in the deep sea, especially for supporting search-and-rescue tasks, where AUVs have to surface frequently to transmit sensory data or events to surface station(s) [35]. Since AUV resurfacing may take quite a long time, sensory data reporting delays occur, which may not be tolerable for certain applications. Hence, a cooperative AUV trajectory planning mechanism is proposed, where the number and locations of AUV resurfacing events are adjusted for cycles with non-sensing edges. Generating an AUV trajectory plan for potential event locations is the main focus, whereas the problem about whether events occur or not is to be explored further. The use case of this technique is to monitor oil pipes, where potential events may be located along oil pipes, which are stable in the deep sea. When the objects to be monitored change their location, the applicability of this technique is not discussed and to be explored further. Am m-course (monitoring course)-based solution for the detection of underwater events is developed in [21]. Generally, a sensor network can be divided among a set of cycles, where sensor nodes can be located in at least one cycle, and the links between sensor nodes, called edges, may pass through these cycles. Intuitively, m-courses may form a type of acyclic flow network, where cycles can establish a tree structure among sensor nodes for routing sensory data to sink node(s). Therefore, when an event occurs, the event location can be determined according to the cycles of corresponding m-courses. The cycles in m-courses are somehow similar to hop counts, which is used for establishing the routing tree in our technique. Generally, this technique aims to localize events, whereas event source identification, which may be important for response enactment, should be explored further. Since the occurrence of events is unpredictable, the information (*i.e*., sensory data) should be higher in value when they can contribute more to the detection of potential events. This means that sensory data relevant to potential events should be routed to the sink node as soon as possible [36]. To maximize the value of information (VoI), AUVs are adopted for sensory data gathering, and initial paths are planned through an integer linear programming model. A distributed heuristic is proposed for path planning online, where the AUV chooses the next sensor node to be visited based on VoI. Generally, this technique aims to gather and route sensory data, which facilitate the event identification, to the sink node with a higher priority. However, energy efficiency, event coverage and source determination are not the focus. To summarize, although event coverage detection has been explored relatively extensively in terrestrial WSNs, there are few techniques addressing this challenge in underwater WSNs nowadays. This article proposes a technique for localizing events in the underwater environment and aims to identify potential event sources for supporting the response enactment.

Traditional techniques on query processing may facilitate the event coverage detection in underwater WSNs. In [22], a sub-region query processing mechanism is proposed, since a sub-region, rather than the whole network region, may be of interest for certain applications. Whether a sensor node is located within an interesting sub-region or not is determined through underwater localization techniques [23]. A routing tree is constructed where hop counts are adopted to represent the distance for sensory data routing to sink node(s). The energy efficiency on routing tree maintenance is of importance since sensor nodes may drift along the water dynamics. Query processing is effective and more energy efficient when multiple queries are issued by users concurrently, and the results of some queries can be reused for answering other queries. Leveraging this observation, the authors proposed a multiple query result merging scheme for reducing the energy consumption in underwater sensory data transmissions [37]. Specifically, queries are rewritten as their simplest forms, which are easier for conducting the queries. When the results of these queries are returned to *SN*, they can be reused for queries with the same data fields and relations. It is argued that this scheme can decrease redundant message transmissions and reduce the energy consumption. In fact, our event coverage detection technique is complementary to query processing mechanisms, which aim to gather and route sensory data packets to sink node(s) in an energy-efficient manner.

Event coverage detection is related to the problem of network boundary determination. In fact, they are similar somehow when events occur in the whole network region. A boundary detection mechanism in three-dimensional wireless networks has been proposed in [18], where sensor nodes on the boundaries are identified based on local information within a one-hop neighborhood. An algorithm that locally constructs planarized triangular meshes is developed and extended from two-to three-dimensions for producing the boundary surface. This work is interesting and inspiring for us to develop the technique in this article. Note that this technique applies to well-connected networks, where no degenerated line segments exist. Specifically, given a line segment between two sensor nodes ($uv_{i}$ and $uv_{j}$), there must be at least one node from which the distances to $uv_{i}$ and $uv_{j}$ are less than the distance between $uv_{i}$ and $uv_{j}$. Due to the sparsity of sensor nodes to be deployed in underwater WSNs, the constraint of well-connected networks may not be satisfied. The network coverage quality is studied with respect to the number of sensor nodes deployed in underwater WSNs [38]. Sensor nodes are deployed at the seabed initially and can move vertically in three dimensions for adjusting their depths and locations. An optimal network coverage can be achieved through the adjustment of sensor nodes, when the coverage can hardly be improved any further. Underwater sensor node deployment for guaranteeing optimal monitoring quality is a challenge in underwater UWSNs [39]. To solve this problem, a depth adjustment algorithm based on a connected tree is proposed, where the parent-child relations specified in the connected tree are adopted for the maintenance of network connectivity and the detection and optimization of network coverage. A connected tree is similar to our routing tree somehow. It is observed that AUVs are increasingly used for the monitoring of vast ocean space. The network coverage should address two problems, including the complete coverage of the whole network region and the connectivity of sensor nodes [40]. Besides, a distributed, rather than a central, controller should be adopted as the guide of the AUVs’ movement. Therefore, this approach enables AUVs to autonomously decide on and adjust their speed and direction at each step, and the global average neighborhood degree is used as the upper limitation of the number of neighbors of each AUV, for achieving a global optimization. To summarize, traditional techniques for detecting the network boundary may be applied for event coverage detection. However, event source identification, which is of importance for proper response enactment, is out of the scope of these techniques. Besides, events may evolve (quickly) due to the dynamics of the underwater environment, so detecting event coverage is a more challenging task in this context. We argue that techniques for network boundary determination can hardly be used directly for determining event coverage.

Event coverage detection and location determination have been studied for two- and three-dimensional terrestrial WSNs. In [41], the topological convex hull of an event region in two-dimensional WSNs is generated in a distributed manner, where no reference and location information or pre-knowledge about the region is required. Sensor nodes report the detection of possible events in a binary mode (one or zero), and sensory data are not gathered. Similarly, [20] proposes a distributed algorithm for the detection of event boundary nodes. Event detection with various user accuracy requirements depends on the specific deployment of sensor nodes and clusters [42]. An event detection method called watchdog is proposed, which can choose and adjust the right energy-efficient sentinel sensor clusters according to certain accuracy requirements. To increase the accuracy and robustness of event coverage, the sensing range of sensor nodes is adjusted at the deployment and event detection phases [43]. Specifically, sensor nodes are one-coverage when deployed and are *k*-coverage for improving the accuracy of event detection. A virtualization for representing event sources is presented in [44], which serve as Internet sources and can be accessed by Internet applications. A survey about the anomaly detection in WSNs is presented in [19]. To summarize, these techniques have inspired us to develop our technique in underwater WSNs. They mainly investigate the problem of event detection and coverage determination, while potential event source identification is not studied extensively, which is one of our contributions in this article. Besides, sensor nodes in underwater WSNs are typically deployed very sparely in the network region, and they are dynamic due to the water dynamics. Consequently, we argue that the techniques in terrestrial WSNs can hardly be applied directly in the context of underwater counterparts.

## 7. Conclusions

Due to the vast un-explored ocean space and harsh underwater environments, the importance and difficulty of underwater exploration is well recognized and underwater wireless sensor networks are emerging as a pressing research topic in recent decades. Smart things, or sensor nodes, are deployed for the monitoring of underwater environments and for the detection of possible events. When sensory data of a set of neighboring sensor nodes deviate from a normal sensing range, the occurrence of events is highly possible, whereas that for a single sensor node may correspond to an outlier or error. An appropriate sensor node should be selected as the relay node for gathering and routing sensory data to the sink node (*SN*). Leveraging the geographical locations of sensor nodes provided by underwater localization techniques, the event coverage is determined by *SN*, which is represented as a weighted graph, where the vertices are sensor nodes, and the weight specified upon the edges reflects the extent of sensory data deviating from a normal sensing range. Event sources are determined, which correspond to the barycenters in this graph. Experimental evaluation shows that our technique is more energy efficient, especially when the network topology is relatively steady.
